# F-Shaped Tunnel Field-Effect Transistor (TFET) for the Low-Power Application

**DOI:** 10.3390/mi10110760

**Published:** 2019-11-09

**Authors:** Seunghyun Yun, Jeongmin Oh, Seokjung Kang, Yoon Kim, Jang Hyun Kim, Garam Kim, Sangwan Kim

**Affiliations:** 1 Department of Electrical and Computer Engineering, Ajou University, Suwon 16499, Korea; shsk5557@ajou.ac.kr (S.Y.); criminal@ajou.ac.kr (J.O.); poorknights@ajou.ac.kr (S.K.); 2 School of Electrical and Computer Engineering, University of Seoul, Seoul 02504, Korea; yoonkim82@uos.ac.kr; 3 Inter-university Semiconductor Research Center, Department of Electrical and with the Department of Computer Engineering, Seoul National University, Seoul 08826, Korea; neuburg@naver.com; 4 Department of Electronic Engineering, Myongji University, Yongin 17058, Korea; garamkim@mju.ac.kr

**Keywords:** band-to-band tunneling, tunnel field-effect transistor (TFET), L-shaped TFET, line tunneling, electric field crowding, corner effect

## Abstract

In this report, a novel tunnel field-effect transistor (TFET) named ‘F-shaped TFET’ has been proposed and its electrical characteristics are analyzed and optimized by using a computer-aided design simulation. It features ultra-thin and a highly doped source surrounded by lightly doped regions. As a result, it is compared to an L-shaped TFET, which is a motivation of this work, the F-shaped TFET can lower turn-on voltage (*V*_ON_) maintaining high on-state current (*I*_ON_) and low subthreshold swing (*SS*) with the help of electric field crowding effects. The optimized F-shaped TFET shows 0.4 V lower *V*_ON_ than the L-shaped TFET with the same design parameter. In addition, it shows 4.8 times higher *I*_ON_ and 7 mV/dec smaller average *SS* with the same *V*_ON_ as that for L-shaped TFET.

## 1. Introduction

**Figure 1:**
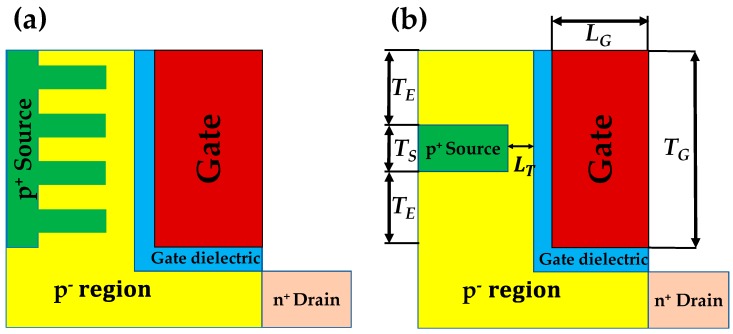
(**a**) Structure of the F-shaped tunnel field-effect transistor (TFET) with multiple sources. (**b**) Structure and parameter definitions of the F-shaped TFET.

Tunnel field-effect transistor (TFET) has been regarded as a promising candidate to replace the metal-oxide-semiconductor FET (MOSFET) for a low power device because its subthreshold swing (*SS*) can be scaled less than 60 mV/dec [1,2,3,4,5,6,7,8]. However, Si-based TFET suffers from low-level on-state current (*I*_ON_) due to its limited band-to-band tunneling (BTBT) rate. Furthermore, there are just a few reports which have demonstrated sub-60 mV/dec *SS* with the experimental devices. Several strategies have been proposed to address these issues [9,10,11,12,13,14,15,16,17,18,19]. Among them, L-shaped TFET has efficiently improved *I*_ON_ and *SS* by increasing BTBT junction area and by decreasing BTBT barrier width (*W*_TUN_) with the help of a novel structure [20]. In spite of these advantages, there is a drawback that turn-on voltage (*V*_ON_), which is defined as gate voltage (*V*_GS_) when BTBT starts to occur, becomes much higher than conventional TFET. It is contradictory to apply the low-power logic elements [21,22]. Therefore, in this manuscript, a new-structure TFET is proposed to address the technical issue of L-shaped TFET maintaining its advantages. Figure 1a shows a schematic structure of proposed device named ‘F-shaped TFET’ because the shape of source is similar to the fingers. It resembles an L-shaped TFET except the ultra-thin sources which are surrounded by intrinsic (or lightly doped) Si regions [20]. It is expected that the F-shaped TFET can reduce *V*_ON_ with the help of electric field crowding effect as the thickness of source (*T*_S_) gets thinner. In order to examine the electrical characteristics of F-shaped TFET, technology computer-aided design (TCAD) simulation is performed [23]. Nonlocal BTBT, Shockley–Read–Hall recombination, bandgap narrowing, and concentration-dependent mobility models are considered for an accurate examination. Table 1 shows the parameters used for the simulation. Gate length (*L*_G_) is set by 20 nm and drain regions are lightly doped to suppress ambipolar behavior.

This manuscript is composed as follows. First, the electrical performance of the F-shaped TFET with a single-source region (i.e., one finger) is examined (Figure 1b). Many parameters such as *T*_S_, lateral length of tunnel region (*L*_T_), and space above and below source (*T*_E_) have been set as variables. In Section 2, the influences of *T*_S_, *L*_T_, and *T*_E_ have been discussed. In Section 3, feasibility for the better performance with F-shaped TFET is examined by adding one more source region (i.e., two fingers) and its design is optimized by adjusting the distance between two source regions (*T*_I_). In Section 4, the optimized design is compared with the conventional L-shaped TFET. In Section 5, an exemplary process flow for the fabrication of F-shaped TFET is proposed.

## 2. Influences of Design Parameters

### 2.1. Length of Tunnel Region (L_T_)

**Figure 2:**
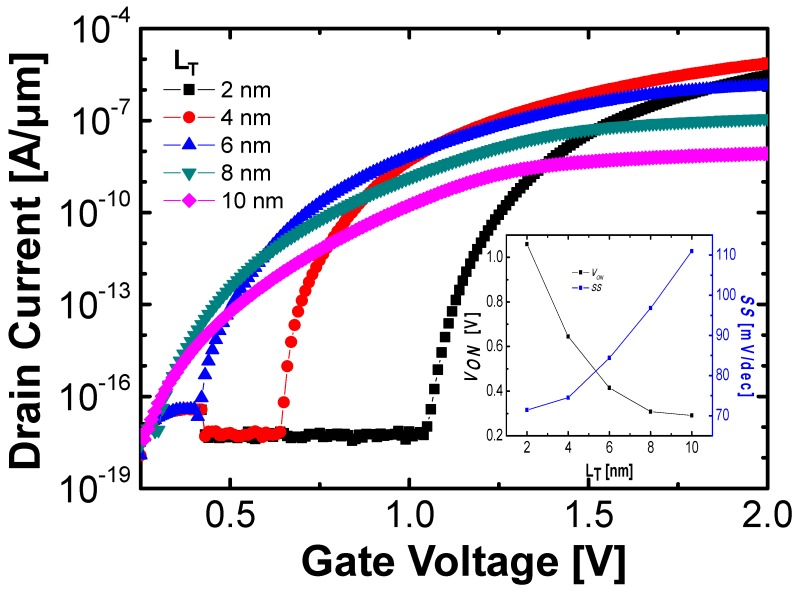
Log scale transfer characteristics with various *L*_T_ at 0.7 V-drain voltage (*V*_DS_). The inset figure shows turn-on voltage (*V*_ON_) and average *SS* (*SS*_AVG_) which is extracted by measuring *SS* from *V*_ON_ to *V*_ON_ + 0.7 V.

Figure 2 shows transfer characteristics as *L*_T_ changes from 10 to 2 nm. It shows that *V*_ON_ increases as *L*_T_ decreases. This is explained by the surface potential depending on *L*_T_ with the help of the voltage division model in series-connected capacitors [24]. In detail, if *L*_T_ decreases, the surface potential at the fixed *V*_GS_ is reduced because the capacitance of the fully depleted Si tunnel region increases; results in a high *V*_ON_. On the other hand, the average *SS* (*SS*_AVG_) decreases if *L*_T_ decreases (Figure 2 and its inset). It is attributed to the smaller *W*_TUN_ (at *V*_GS_ = *V*_ON_) with the smaller *L*_T_ [24]. Similarly, *I*_on_ increases as *L*_T_ decreases, because the *W*_TUN_ at on-state decreases. The optimum *L*_T_ is determined as 4 nm, since the increase of *V*_ON_ is significant while the reduction of *SS*_AVG_ is negligible as *L*_T_ becomes less than 4 nm (inset of Figure 2).

### 2.2. Source Thickness (T_S_)

**Figure 3:**
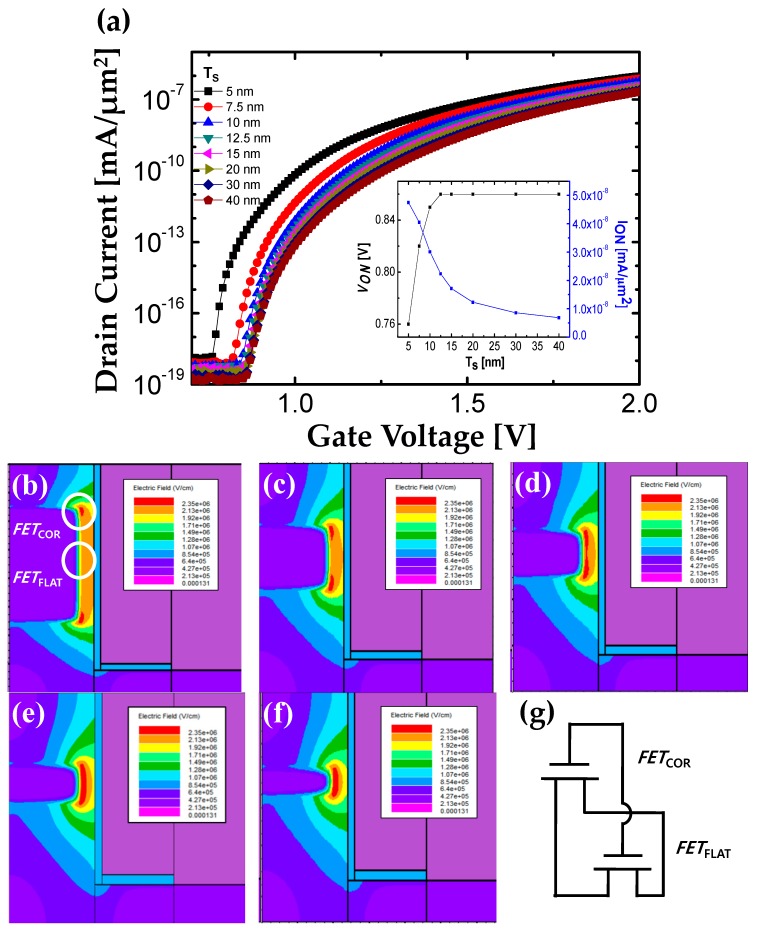
(**a**) Normalized log scale transfer characteristics with various *T*_S_ at *V*_DS_ = 0.7 V. The inset figure shows *V*_ON_ and normalized on-state current (*I*_ON_) which is defined as *I*_D_ at *V*_GS_ = 0.5 + *V*_ON_ divided by *T*_S_. Electric field contour plots for (**b**) *T*_S_ = 40 nm, (**c**) *T*_S_ = 15 nm, (**d**) *T*_S_ = 10 nm, (**e**) *T*_S_ = 7.5 nm, and (**f**) *T*_S_ = 5 nm. These plots are extracted at *V*_DS_ = 0.7 V and *V*_GS_ = 0.86 V which is corresponded to the *V*_ON_ of *T*_S_ = 40 nm. (**g**) Schematic circuit model for F-shaped TFET.

Figure 3a shows transfer characteristics depending on *T*_S_. The drain current (*I*_D_) is normalized by *T*_S_ to exclude the influence of *T*_S_ on the BTBT junction area and on the magnitude of *I*_D_. There are two noteworthy points in terms of *I*_ON_ and *V*_ON_ as shown in the inset of Figure 3a. Both results can be analyzed by electric field contour plots shown in Figure 3b–f. As shown in Figure 3b, electric field at sharp source corner (*E*_COR_) is much larger than that for flat source region (*E*_FLAT_) due to field crowding effect [25]. Because *V*_ON_ and *I*_ON_ of TFETs sensitively depend on electric field at source-to-channel junction, the source corner and the flat source regions can be regarded as different TFETs; *FET*_COR_ and *FET*_FLAT_. In other words, F-shaped TFET can be regarded as *FET*_COR_ and *FET*_FLAT_ connected in parallel as shown in Figure 3g. If *T*_S_ decreases, the *FET*_COR_ contributes more to *I*_D_ than *FET*_FLAT_. As a result, the normalized *I*_D_ by *T*_S_ is increased because *FET*_COR_ has higher current than *FET*_FLAT_.

Unlike to *I*_ON_*, V*_ON_ is solely determined by *FET*_COR_ which is turned on first. Although *T*_S_ decreases (i.e., the portion of *FET*_COR_ increases), the *E*_COR_ is unchanged. Therefore, *V*_ON_ is not affected by *T*_S_ from 40 to 10 nm (Figure 3b–d). On the other hand, if *T*_S_ becomes less than 10 nm, *FET*_FLAT_ is completely disappeared and *FET*_COR_ at two corners starts to be merged (Figure 3e,f). As a result, the magnitude of electric field is increased further and *V*_ON_ starts to be decreased. Considering process capability, *T*_S_ is optimized as 5 nm.

### 2.3. Space Above and Below Source (T_E_)

**Figure 4:**
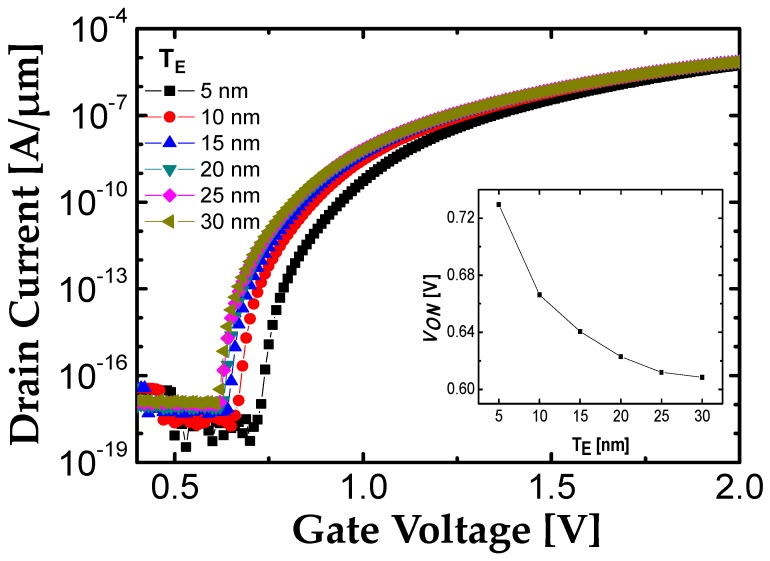
Transfer characteristic with the various *T*_E_ at *V*_DS_ = 0.7 V. The inset figure shows extracted *V*_ON_ with the variation of *T*_E_ ranging from 5 to 30 nm.

As discussed in Figure 1, unlike the L-shaped TFET, the source of the F-shaped TFET is surrounded by lightly doped Si regions. Therefore, it is worthwhile to study about the influence of *T*_E_ on electrical characteristics of F-shaped TFET because it can influence on electric field crowding. As shown in Figure 4, the *V*_ON_ slightly decreases as the *T*_E_ increases due to the increase of electric field crowding effect. In other word, the number of electric field flux is increased since the tunnel junction is affected by the larger gate area. Consequently, band bending at tunnel junction becomes abrupt, and hence decreases *V*_ON_. However, large *T*_E_ is contradictory to the process capability (i.e., abrupt etching profile). In addition, if *T*_E_ increases more than 15 nm, the decrease of *V*_ON_ is negligible as shown in the inset of Figure 4. Based on the above results, *T*_E_ is optimized as 15 nm.

## 3. Optimized F-Shaped TFET

**Figure 5:**
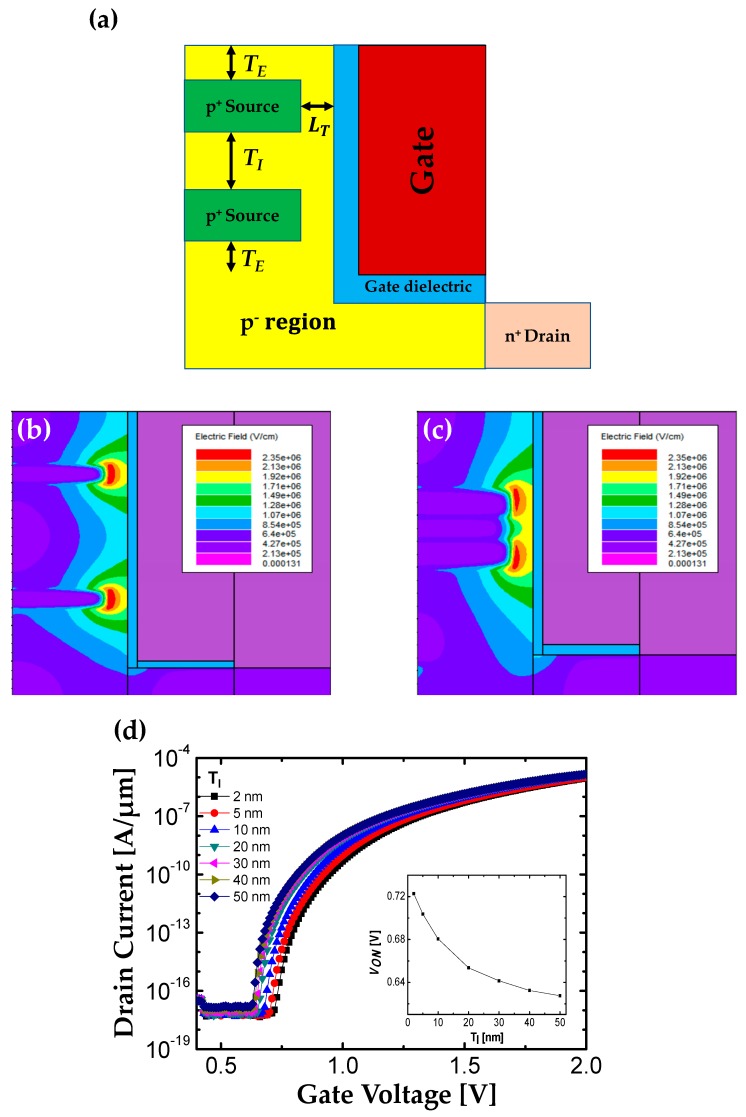
(**a**) Structure of F-shaped TFET with multiple sources. Electric field contour plots for (**b**) *T*_I_ = 30 nm and for (**c**) *T*_I_ = 5 nm at *V*_DS_ = 0.7 V, *V*_GS_ = 0.86 V. (**d**) Transfer characteristic as *T*_I_ increases from 2 to 50 nm at *V*_DS_ = 0.7 V. The inset figure shows extracted *V*_ON_.

In Section 2, the parameters (*T*_S,_ *L*_T_, *T*_E_) which can influence on the electric field crowding effect have been optimized by several simulations. Although F-shaped TFET can achieve the higher normalized *I*_D_ (i.e., current density) as *T*_S_ decreases, the smaller BTBT junction area is problematic in terms of total current for its real application. It can be addressed by adding an additional source (i.e., figure) as shown in Figure 5a. From the previous results in Section 2.3, it can be expected that the electrical characteristic of F-shaped TFET with multiple source regions is sensitively affected by the distance between the two sources (*T*_I_). Therefore, the influences of *T*_I_ on the electrical performance of F-shaped TFET are investigated to determine an optimum *T*_I_. Figure 5b,c shows the effect of *T*_I_ on the magnitude of the electric field at source-to-channel junction. If *T*_I_ gets smaller, the electric field of both sources start to become merged and each electric field at tunnel junction is decreased. As a result, *V*_ON_ is increased as shown in Figure 5d and its inset. The result is well corresponded to the phenomena discussed in Section 2.3. Considering the process capability and the influence of *T*_I_ on the electrical performance, *T*_I_ is optimized as 30 nm.

## 4. Comparison with L-Shaped TFET

**Figure 6:**
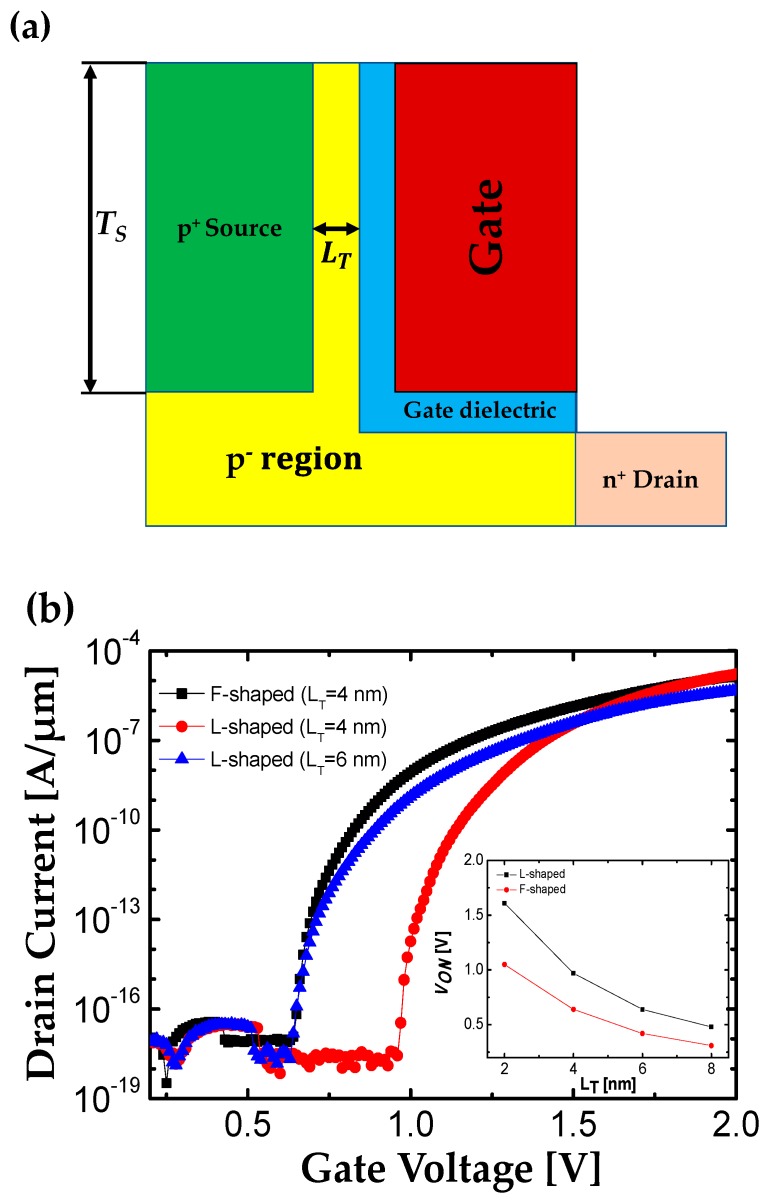
(**a**) Structure of L-shaped TFET. (**b**) Transfer characteristics of L-shaped and F-shaped TFETs at *V*_DS_ = 0.7 V. The inset figure shows *V*_ON_ of both TFETs as a function of *L*_T_ from 2 to 8 nm.

Figure 6a shows a schematic structure of L-shaped TFET studied in [24]. Most of design parameters such as *L*_G_, *T*_OX_, *N*_S_, *N*_D_, *N*_B_, *W*_FN_ and *W* are the same as that for the F-shaped TFET. In case of L-shaped TFET, *T*_S_ is set by 70 nm which is the same as *T*_G_ in optimized F-shaped TFET; *T*_S_ = 5 nm, *T*_E_ = 15 nm, and *T*_I_ = 30 nm, *T*_G_ = 2*T*_S_ + 2*T*_E_ + *T*_I_ (Figure 5a). On the other hand, *L*_T_ is set as 4 nm or 6 nm to compare with F-shaped TFET in two points of view; the same dimension or *V*_ON_.

In case of 4 nm-*L*_T_, L-shaped TFET has the same dimension as the optimized F-shaped TFET discussed in Section 2.1. As shown in Figure 6b, it is clear that the *V*_ON_ of F-shaped TFET is about 0.4 V lower than that for L-shaped TFET in spite of the same dimension with the help of the electric field crowding effect. The inset of Figure 6b confirms that *V*_ON_ of F-shaped TFET is always smaller than that for L-shaped TFET with the same *L*_T_.

If the *L*_T_ of L-shaped TFET is 6 nm, its *V*_ON_ becomes the same as that of an optimized F-shaped TFET (Figure 6b). Comparing both TFETs with the same *V*_ON_, the *I*_ON_, and *SS*_AVG_ of F-shaped TFET are 4.8 times higher and 7 mV/dec lower than that for L-shaped TFET, respectively. The results are clearly attributed to the enhanced BTBT rate with the geometrical merits (i.e., field crowding), because F-shaped TFET has smaller BTBT junction area than L-shaped TFET.

## 5. Device Fabrication

**Figure 7:**
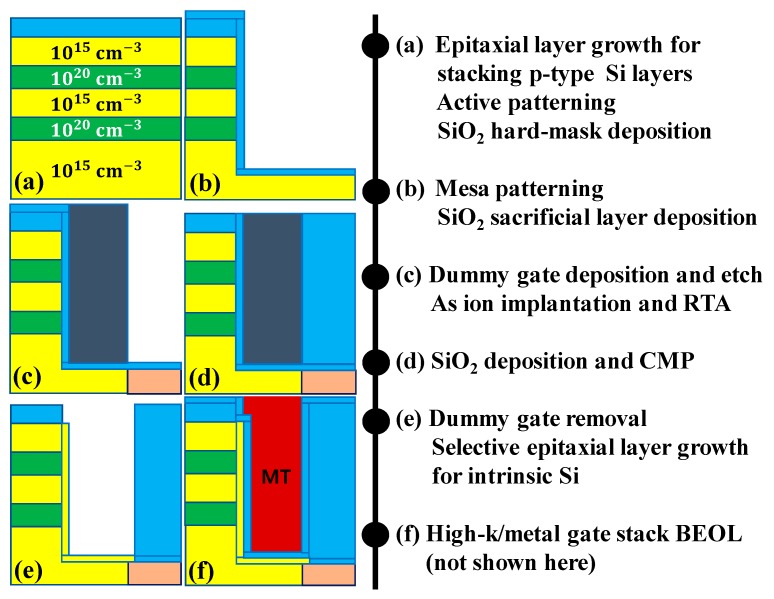
An exemplary process flow for the fabrication of an F-shaped TFET.

Figure 7 summarizes an exemplary self-align process flow for F-shaped TFET with multiple source regions; fingers. (Figure 7a) P-type Si layers doped by 10^20^ cm^−3^ and 10^15^ cm^−3^ are alternately stacked on Si-on-insulator (SOI) wafer through epitaxial layer growth processes. After defining an active region, SiO_2_ hard-mask is deposited by a chemical vapor deposition (CVD). This layer is also helpful to passivate active sidewall. (Figure 7b) Mesa patterning is followed by SiO_2_ buffer layer deposition. (Figure 7c) After dummy gate formation by deposition and etch-back processes, drain region is defined by arsenic (As) ion implantation and rapid thermal annealing (RTA). (Figure 7d) SiO_2_ deposition is followed by chemical-mechanical polishing (CMP) to expose the dummy gate. (Figure 7e) After selectively removing the dummy gate and SiO_2_ buffer layer, selective epitaxial layer growth (SEG) is performed to form ultra-thin tunnel region. (Figure 7f) The gate stack is formed by high-k/metal gate atomic layer deposition (ALD) processes. The back-end-of-line process is not shown here.

## 6. Summary

A novel F-shaped TFET is proposed and its device physics and operating mechanisms are studied in detail by using two-dimensional TCAD simulations. The results confirm that it can achieve a relatively lower *V*_ON_ (~0.6 V) than that for L-shaped TFET (~1.0 V) with the same *L*_T_. In addition, the current drivability of F-shaped TFET can be further improved by adding additional sources (fingers). Last of all, F-shaped TFET is expected to be fabricated by self-aligned processes. Therefore, F-shaped TFET can be regarded as one of the promising candidates for low-power digital logic applications.

## Figures and Tables

**Table 1:** Parameters used for the technology computer-aided design (TCAD) simulation.

Abbreviations	Parameter	Value
*L*_G_	Gate length	20 nm
*L*_T_	Lateral length of tunnel region	Variable
*T*_OX_	Gate oxide thickness	2 nm
*T*_G_	Gate thickness	2*T*_E_ + *T*_S_
*T*_E_	Space above and below source	Variable
*T*_S_	Source thickness	Variable
*N*_S_	P-type source doping concentration	10^20^ cm^−3^
*N*_D_	N-type drain doping concentration	10^18^ cm^−3^
*N*_B_	P-type body doping concentration	10^15^ cm^−3^
*W*_FN_	Gate work function	4.05 eV
*W*	Channel width	1 μm
